# C4-HSL aptamers for blocking qurom sensing and inhibiting biofilm formation in *Pseudomonas aeruginosa* and its structure prediction and analysis

**DOI:** 10.1371/journal.pone.0212041

**Published:** 2019-02-19

**Authors:** Meng Zhao, Weibin Li, Kuancan Liu, Huiling Li, Xiaopeng Lan

**Affiliations:** 1 Second Military Medical University, Shanghai, China; 2 Institute for Laboratory Medicine, The 900th Hospital of Joint Service Support Force, Fuzhou, Fujian, China; Laurentian, CANADA

## Abstract

This study aimed to screen DNA aptamers against the signal molecule C4-HSL of the rhl system for the inhibition of biofilm formation of *Pseudomonas aeruginosa* using an improved systematic evolution of ligand by exponential enrichment (SELEX) method based on a structure-switching fluorescent activating bead. The aptamers against the C4-HSL with a high affinity and specifity were successfully obtained and evaluated in real-time by this method. Results of biofilm inhibition experiments in vitro showed that the biofilm formation of P. aeruginosa was efficiently reduced to about 1/3 by the aptamers compared with that of the groups without the aptamers. Independent secondary structure simulation and computer-aided tertiary structure prediction (3dRNA) showed that the aptamers contained a highly conserved Y-shaped structural unit. Therefore, this study benefits the search for new methods for the detection and treatment of *P*. *aeruginosa* biofilm formation.

## Introduction

*Pseudomonas aeruginosa* (*P*. *aeruginosa*) is a common opportunistic pathogen in the human body. *P*. *aeruginosa* is the main pathogen that causes the bacteremia in patients with burn injury, catheter-associated urinary tract infection or ventilator-acquired pneumonia [1,2]. Immunocompromised patients such as cancerous patients or bone marrow transplant patients are extremely easily infected by this pathogen. The death rate of the ventilator-acquired pneumonia induced by this strain in patients with endotracheal intubation can reach as high as 38% [3]. Furthermore, in the growing cases of AIDS patients, 50% of the death is related to the *P*. *aeruginosa* bacteremia [4,5]. In addition, *P*. *aeruginosa* is the primary cause of morbidity and mortality of patients with cystic fibrosis patients [6–8]. Compared with other pathogens, *P*. *aeruginosa* is difficult to eradicate owing to its intrinsic resistance to numerous antibiotics, such as aminoglycosides, fluoroquinolones, and beta lactams. As *P*. *aeruginosa* can generate biofilm on the inner surface of the physiological cavities or pipelines, such as the respiratory tract and sinus cavity, it causes refractory infection and delay of complete recovery. Therefore, efficient inhibition of the biofilm formation of *P*. *aeruginosa* is a promising way to defend against the infection by this pathogen [9,10].

Aptamers are in vitro chemically synthesised oligonucleotides with high specificity and sensitivity toward a specific target. Aptamers feature advantages over antibodies as they possess good thermal stability, permit easy introduction of chemical modification, and can be easily produced by chemical synthesis. Given these advantages, aptamers are increasingly gaining traction as molecular recognition elements of biosensors and in medical applications [11,12].

Quorum sensing (QS) plays an important role in the formation of P. aeruginosa biofilms. Under the control of QS, the bacteria communicate with each other via signals, and then coordinate certain behavior to resist pressure from the external environment [13, 14]. Currently, there are three QS systems exist in *P*. *aeruginosa*: las system (lasI/lasR), rhl system (rhlI/rhlR), and Pseudomonas quinolone signal system (PQS). The rhl system of *P*. *aeruginosa* contains rhlI and rhlR genes. The former gene encodes an acylhomoserine lactone (AHL) synthase for the biosynthesis of N-butanoyl-homoserine (N-C4-HSL), which is a small molecular compound that can freely penetrate cell walls and cell membranes, and the latter encodes the regulator for the transcription of numerous virulence factor genes [15–18]. The three QS systems mentioned above are closely related to the *P*. *aeruginosa* biofilm formation. Previous studies showed that deficiency in the QS-relevant genes could dramatically reduce the biofilm formation and drug resistance of the *P*. *aeruginosa* [19].Both the las system and rhl system could notably affect the formation and maintenance of the biofilm [20–21]. Therefore, depressing the rhl system shows promise in disturbing QS of the bacteria and subsequently inhibit the biofilm formation by depressing the rhl system. In this study, the structure-switching SELEX (systematic evolution of ligand by exponential enrichment) method was designed to screen aptamers with high affinity and high specificity against the signal molecule C4-HSL of the rhl system. As an inhibitor of the QS system in *P*. *aeruginosa*, aptamers can specifically bind to the C4-HSL secreted by the bacteria, causing the signaling molecule to be maintained at a low levels in the culture or prohibiting the rhl system from participating in the QS regulation by blocking the conjugation site between the signaling molecule and the rhlR protein. As expected, QS was disturbed and biofilm formation was inhibited by the aptamers. In this study, the structure of the specific aptamers against C4-HSL of the *P*.*aeruginosa* was also predicted and analyzed.

## Materials and methods

### *P*. *aeruginosa*

The wild-type(wt) *P*. *aeruginosa* strain used in this study was isolated from clinical infection patients. The *⊿rhlI/⊿lasI P*. *aeruginosa-*deficient strain, which was first isolated by the Hospital of Copenhagen University, was a kind gift provided by Dr. Lu Qi of Children's Hospital of Chongqing Medical University. LumAvidin microspheres coated with Avidin, which could ligate to the biotinylated C50, were purchased from Luminex Corp(Austin, TX). C4-HSL was purchased from Cayman company.

### Reagents for SELEX screening

LumAvidin microspheres coated with Avidin, which can ligate to the biotinylated C50, were purchased from Luminex Corp. (Austin, TX). C4-HSL was dissolved in SELEX buffer and stored in 250 μM storage solution at -20°C. All the buffers for the SELEX screenings were diluted in PBS buffer. A hybridization buffer (1 mM PB, 1 mM NaCl, 30 mM MgCl, and pH = 6.9) was used in the hybridization of Pool_99_ and capturing the sequence of CD50. The assembling-washing buffer (150 mM PB, 1 M NaCl, 5 mM MgCl_2_, 0.01% Tween-20, 1% BSA, and pH = 6.9) and SELEX buffer (150 mM NaCl, 25 mM Tris-HCl, 25 mM KCl, 20 mM MgCl_2_, and pH = 7.4) were also used in this study.

### Random ssDNA library construction, primers, and capturing sequence synthesis

The random ssDNA library was a 99-nucleotide (nt) length library (Pool_99_). At each end of the ssDNA was a fixed sequence with 18 nt, whereas a fixed hybrid sequence with 15nt was flanked by four thymines in the middle of each side. The four thymines were designed to enhance the flexibility of the ssDNA. Between every 4 thymines and each end were 30 and 10 random nt. The sequence of the ssDNA was designed as 5ʹ-CCATCCACACTCCGCAAG-N30-TTTT-hybridsequence-TTTT-N10-CGTCGGCTGCCTCTACAT- 3ʹ. The capacity of the library was more than 10^24^ (4^40^). The forward primer was 5ʹ-CCATCCACACTCCGCAAG-3ʹ and the reverse primer was 5ʹ-ATGTAGAGGCAGCCGACG-3ʹ corresponding to the fixed sequences at each end of the ssDNA for amplifying dissociated ssDNA using PCR. The library and primers were supplied by Shengong Biotechnology Co. Ltd. (Shanghai, China). The capturing sequence was reverse complementary to the hybrid sequence, and Cy3 fluorophore occupies the 5ʹ side. In the 3ʹ end of the capturing sequence, 15 nt thymines, 6 carbon atoms, and a biotin group were sequentially connected. The quenching sequence SQ was the same as the reversed primer but with a quencher BHQ-1 labeled at the3ʹ end.

### Hybridization of C50, Pool_99_, and SQ

First, the ssDNA library (Pool_99_) was dissolved in the assembling-washing buffer and was denatured at 95°C for 5 min. Then the library was placed on ice water as soon as possible to cool down to 0°C. The cooled library was mixed with C50 and S_Q_ at a ratio of C50:Pool99:SQ = 6:1:6. The final volume of this mixture was not more than 20 μL. The mixture was immediately placed in water at 53°C for 30 min, followed by another water immersion at 48°C for more than 4 h. Next, the mixture was held in water at 43°C for 30 min and 38°C for 4 h.

### Assembling the Bead-C50-Pool_99_-SQ complex

LumAvidin microspheres stored at the low temperature were removed and warmed to room temperature, centrifugated and ultrasonically shaken, and completely resuspended. The acquired volume of the microspheres was transferred into a 1.5 mL low-protein-adsorption tube (Eppendorf) and centrifugated. Then, the mixture was added to 500 μL of the assembling-washing buffer and gently vortexed to resuspend the microspheres, and centrifugated twice. The hybridized C50-Pool_99_-SQ was added to the LumAvidin microspheres that had been washed. Then the complex Bead-C50-Pool_99_-SQ was completely assembled. The microspheres were then sequentially washed by the hybrid-washing buffer with different concentrations of NaCl at 500, 100, 50, 20 and 1 nM. The procedure was as follows: shaking at 400 rpm for 30 min each time, and thrice for every NaCl concentration. The supernatant of the washed buffer was kept every third time for the following PCR to evaluate the effect of washing.

### Screening the C4-HSL aptamer by the structure-switching fluorescence activating bead sorter (ssFABS)

**Fig 1:**
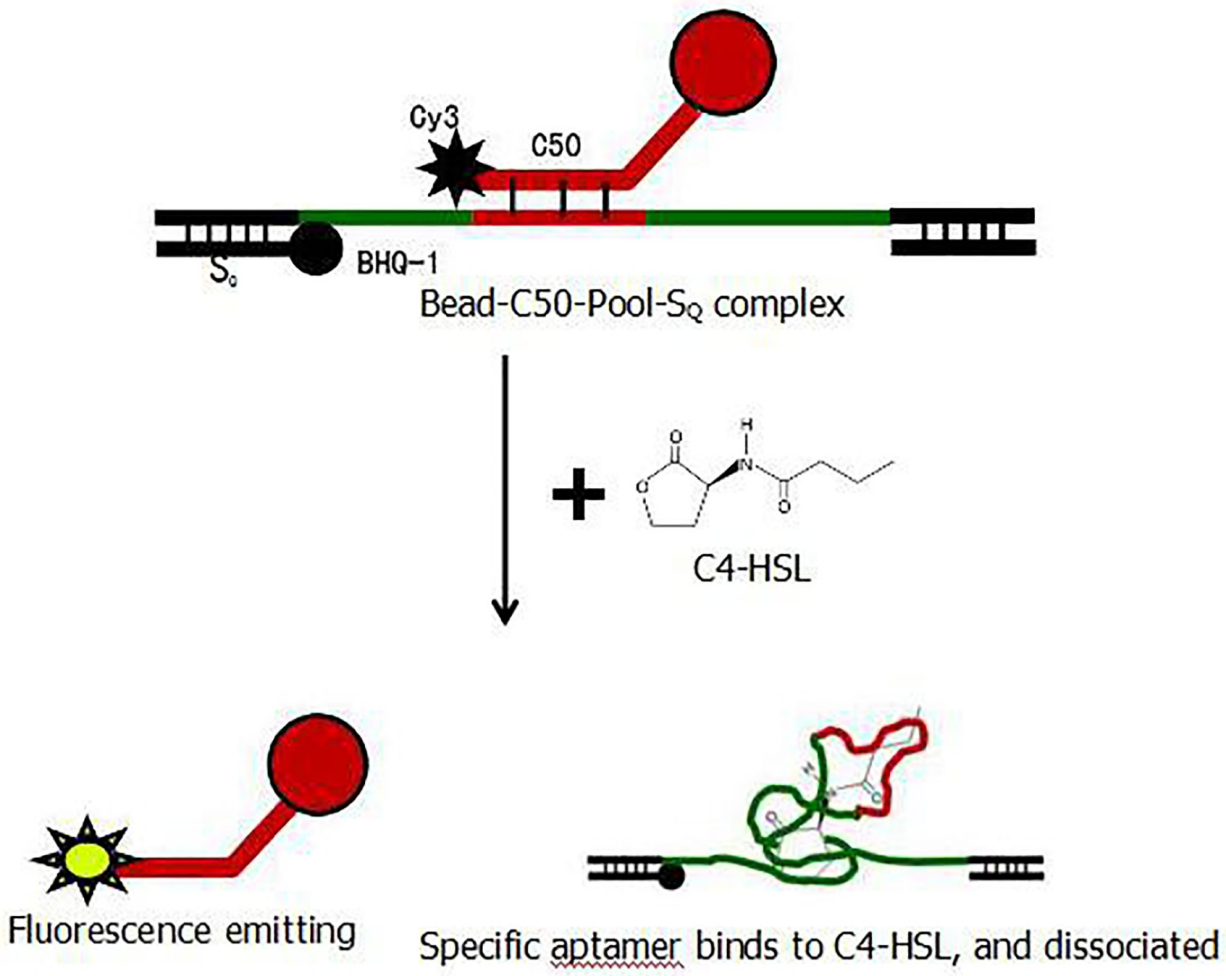
Schematic of aptamer screening by structure switching.

As the signaling molecule C4-HSL is a small compound with a MW of 170, it is difficult to be fixed on a solid matrix. Therefore, the immobilized aptamer method was used to screen the specific aptamer. In this method, the fixed aptamer changes its conformation after it binds to C4-HSL, and it then dissociates from the solid phase into the liquid phase (Fig 1). The structure-switched aptamer in the liquid phase can be directly used in PCR for amplification, and the product can be subsequently applied in the next round of screening. With rounds of screening, aptamers with a high affinity and specificity can be obtained. The method above is called the structure-switching SELEX method.

### Preparation and purification of the C4-HSL structure-switching ssDNA

The Bead-C50-Pool99 complex prepared above was resuspended in SELEX buffer. C4-HSL was added to the complex and incubated together at 37°C for 1 h for conformation transition. Then, the mixture was centrifugated at 8,000 g for 2 min, and the supernatant was collected. A portion of the supernatant was used for PCR to measure the switching rate, and the rest was kept for the following structure switching. Asymmetric PCR was used to obtain the secondary ssDNA library. The PCR protocol was implemented at an initial temperature of 95°C for 5 min, followed by 40 cycles of denaturing at 95°C for 30 s, annealing at 60°C for 30 s, extension at 72°C for 15 s, and one cycle of extension at 72°C for 4 min. The PCR product was isolated and purified by the Spin Column DNA Gel Extraction Kit for PAGE and was used as the secondary ssDNA library for the next round of screening.

### Structure switching SELEX evolution

The random ssDNA library used in the first round was 1 OD,and about 1.1 nmol ssDNA was used for the assembly of the Bead-C50-Pool_99_-SQ complex. C4-HSL was used at a final concentration of 25 μM. After structure switching for 3 h at 30°C, the supernatant was used in the pre-PCR, and the remainder was kept overnight for switching. From the second round, the amount of ssDNA used, together with the proportion of target molecules to ssDNA, was progressively decreased, and the reaction temperature was gradually decreased to room temperature with the reaction time shortened.

The efficiency of each screening round was calculated as Rsw using the following formula: *R*_*swn*_ = (*F*_*sw*_-*F*_*Q*_) ∕ (*F*_*SB*_-*F*_*Q*_)×(*T* • *min*) ×100%. In this formula, F_Q_ and F_SW_ represent the fluorescence intensity before and after structure switching, respectively. The former was measured immediately after assembling the Bead-C50-Pool_99_-SQ complex. The latter was determined immediately after structure switching with C4-HSL for a certain period of time. F_SB_ represents the fluorescence intensity measured for the control tube without C4-HSL after the same length of time as the switching tube at the same temperature. T•min is used as the denominator to normalize the effects of different lengths of time and different temperatures on each round. Therefore, R_sw_ is defined as the ratio of the fluorescence intensity enhanced from structure switching by C4-HSL to the background fluorescence intensity increase due to the natural dissociation of the complex. When the Rsw growth curve reaches the plateau, the aptamer screening process is completed.

### Cloning, sequencing and analysis of the screened aptamers

When the screening procedure was completed, the final product was amplified by PCR, agarose electrophoresis, dsDNA purification, T-A cloning, and “blue-white” screening. Finally, 59 positive colonies were randomly selected and sequenced.

The primary sequences of all aptamers were analyzed for homology and repeatability using bioinformatics software such as Clustal W and MEGA6.0. The predictions of secondary structures of aptamers were obtained through the searching for a similarly conserved structure by the Multilign method of RNAstructure5.6 software. At last, the software 3dRNA was used to predict the possible tertiary structure of the aptamer.

### Determining the affinity of aptamer by saturation binding experiments

Ensuring that the aptamerwas excessively relative to C4–HSL, the assembled Bead-C50-Pool_99_-SQ complex was equally separated into 7 tubes (labeled 0–6) containing C4-HSL at final concentrations of 0, 25, 50, 100, 200, 400, and 600 nM. At room temperature, after 1 hour of structure switching, fluorescence intensity was measured by Luminex^TM^100,and the aptamer dissociation rate at different C4-HSL concentrations was calculated with the following formula: *R’*_*swn*_ = (*F*_*swn*_-*F*_*0*_) ∕ (*F*_*C50*_*F*_*Q*_)×100%. In this formula, *F*_*0*_ and *F*_*swn*_ represent the fluorescent intensity of tube 0 (the control tube) and tubes 1–6 after structure switching for 1 h, respectively. Thus, *F*_*swn*_-*F*_*0*_ represents the amount of the dissociated aptamers under different concentrations of C4-HSL. *F*_*C50*_ represents the fluorescence intensity of the same amount of C50 captured by the same amount of microspheres. Thus, *F*_C50_-*F*_Q_ can represent the amount of the aptamers that should be assembled on the microspheres. As the amount of aptamer dissociated from the complex is equal to the amount of aptamer bound to C4-HSL, therefore, *R’*_*swn*_ could be used as an indicator of the capability of the aptamer to bind to C4-HSL. Finally, the dissociation constant Kd of the aptamer was calculated by data-fitting with the One Site Bind-Saturation Analysis Model in software Origin 7.5.

## Determining aptamer specificity

The Bead-C50-pool_99_-S_Q_ complex was equally separated into 2 Eppendorf tubes. Then 2 (5H)-furanone was added into the tubes to final concentrations at 400 nM and 600 nM, respectively. The mixture was then placed in a 400 rpm shaker for 1 h for structure switching, and the fluorescent value of the microspheres was measured. The results were compared with those of microspheres treated with same concentration of C4-HSL.

### Bacterial growth inhibiting test by aptamers

The bacterial growth curve with aptamers test was divided into 6 groups (3 tubes in parallel in each group), namely wt, wt+A16, wt+A46, wt+A1, wt+pool99, and lasI/rhlI deficient mutant, which represent the wild-type untreated group, the aptamer 16-treated wild-strain, aptamer 46-treated wild strain, aptamer 1-treated wild strain, random ssDNA library (pool99)-treated wild-strain group, and lasI/rhlI-deficient mutant-untreated groups, respectively. The medium was SB-LB buffer diluted 5 times with SELEX buffer, 3 ml per test tube, which was added in 30 ul the different bacteria suspension with 1.0 Macfarland standard. Then, each test tube was incubated for 7 h at 37°C, 50 rpm. The optical density of 200 μl suspension was measured at a wavelength of 600 nm every 1 h. Finally, the growth curve of each group was drawn and statistically analyzed to explore whether the aptamer inhibits the normal proliferation of bacteria.

### Monitoring the biofilm formation inhibition in vitro through ESEM

The experiment was divided into 4 groups, including the defect strain, wt, wt+pool99 and wt+aptamer groups, corresponding to 4 sterilized glass culture dishes. The solid substrate with growing biofilm growing was a sterilized piece of polyvinyl chloride plastic with a size of 1 cm×1 cm. The liquid medium was SB-LB liquid medium diluted 5 times with SELEX buffer. The final density of the first inoculated bacteria was about 4.8×10^6^ / L. As with pool99, the final concentration of the aptamer was 40 nM. Renew the liquid medium and re-add aptamer or pool99 was re-added to maintain the same concentration every 2 days. After 7 days, the plastic block was removed, washed three times with SELEX buffer, and 20%, 40%, 60%, 80%, and 100% ethanol. Finally, ESEM was used to observe the biofilm (20 KV) after evaporating ethanol to dry at room temperature.

### Quantitative determination of the biofilm by the crystal violet staining method

Quantitative determination of the biofilm was grouped into 6 groups: wt type strain group treated with aptamer 16 (group 1), wt type strain group treated with aptamer 46 (group 2), wt type strain group treated with aptamer 1 (group 3), untreated wt type strain group (group 4), pool99-treated wt strain group (group 5), and deficient mutant groups (group 6). A polystyrene 96 U-well plate was used as a matrix for biofilm formation, and 12 wells were repeated for each group. In groups 1–3 and 5, the final concentration of pool99 or aptamers totaled 40 nM. The 6 mixtures were incubated overnight at 37° C and 50 rpm in a shaker. The next day, the liquid in each well was abandoned, and the wells were washed 3 times with switching buffer, then the medium was re-added, and aptamers or pool99 were added to each well with the final concentration of 40 nM, respectively. Then continue to cultivate in the same shaker. Next, the steps of washing and liquid changing were repeated every 48 hours till cultivated for 7 days. At the end of the cultivation, formaldehyde (99%) was added to each well to fix the films for 15 min after discarding the liquid and dried in the air. A 1.5% crystal violet solution was added to each well and the biofilms were stained for 10 min at room temperature, washed and drying in air. Finally, 95% ethanol (200 μL) was added until decolorization for 3 min. The OD value was measured at 570 nm. Data statisticswere determined using one-way ANOVA.

## Results

### Assembling structure switching of the Bead-C50-Pool_99_ complex

As shown in Table 1, the ratio of the microsphere: capturing-sequence: pool_99_ library used in the first round SELEX was 18:6:1. The assembly rate measurement showed that the rate reached as high as 99.33%. This value implies that almost all of the pool_99_ library was immobilized onto the microsphere, which can effectively ensure the diversity of the random pool for ssFABS-SELEX evolution.

**Table 1:** Assembly rate of Bead-C50-Pool_99_ complex.

	First time	Second time	Third time	Average
Fluorescent value before capture, F0	29991	30074	30002	30022.3
Fluorescent value after capture, F1	195	201	204	200
Assembly rate of the Bead-C50-Pool_99_ complex = (F_1_-F_0_)/F_0_×100% = (30022.3–200)/30022.3 = 99.33%				

### Real-time monitoring of SELEX screening by ssFABS

**Fig 2:**
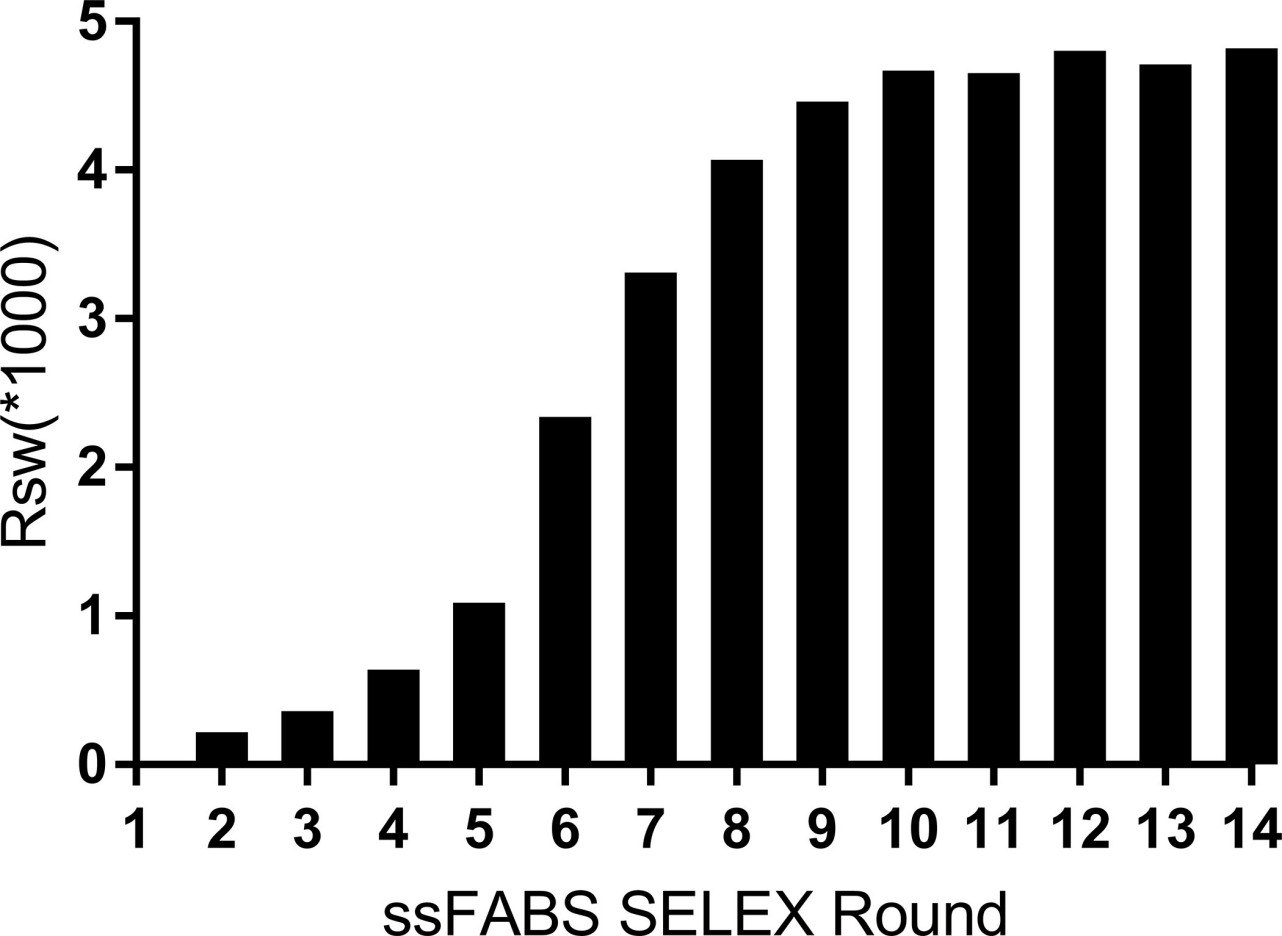
Schematic of aptamer screening by structure switching.

As shown in Table 2, the background of switching was only 1.14%,and the quenching rate was maintained around 60%. The efficiency of SELEX increased rapidly and reached the plateau at the 10th round although the temperature gradually decreased and the length of structure-switching time gradually shortened with the increaseing screening round (Fig 2), indicating that ssFABS is more feasible and efficient for screening aptamers, and can simultaneously monitor the efficiency of the evolution process.

**Table 2:** Monitoring the SELEX screening by ssFABS.

	*F*_*Q*_	*F*_*sw*_	*F*_*SB*_	Temperature (°C)	Time (min)	Quenching rate (%)	SB Structure-switching Rate (%)	R_sw_ (×1000)
2	804.27	834.93	811.24	37	540	59.79	0.87	0.22
3	672.31	722.87	679.27	37	540	60.45	1.04	0.36
4	665.88	713.39	674.28	37	240	60.83	1.26	0.64
5	634.24	693.06	640.33	37	240	57.15	0.96	1.09
6	544.65	651.32	550.97	30	240	59.66	1.16	2.34
7	529.7	729.46	538.08	30	240	60.76	1.58	3.31
8	240.09	280.69	242.86	30	120	65.7	1.15	4.07
9	361.94	421.47	366.22	26	120	63.81	1.18	4.46
10	259.68	307.58	262.97	26	120	59.43	1.27	4.67
11	450.55	520.42	455.37	26	120	60.82	1.07	4.65
12	480.32	548.79	484.89	26	120	62.48	0.95	4.8
13	500.18	563.83	505.96	26	90	60.92	1.16	4.71
14	315.51	341.81	319.01	26	60	59.29	1.11	4.82
Average						60.85	1.14	

### TA cloning, sequencing, and homology analysis

After sequencing and homology analysis, 47 aptamers obtained from 59 positive TA clones, as shown in supplement data, can be roughly divided into 9 families. Among these families, 2, 4, 3 and 3 identical aptamers belong to families IV, VII, IX and III, respectively. (Table 3).

**Table 3:** Repeatability analysis of the family sequence.

No.	Aptamer	Families	Repeated number	Total sequences within family	Repeated proportion
1	A16	F-IV	2	2	100.0%
2	A46	F-VII	4	6	66.7%
3	A1	F-IX	3	5	60.0%
4	A2	F-III	3	5	60.0%

### TA cloning, sequencing, and homology analysis

**Fig 3:**
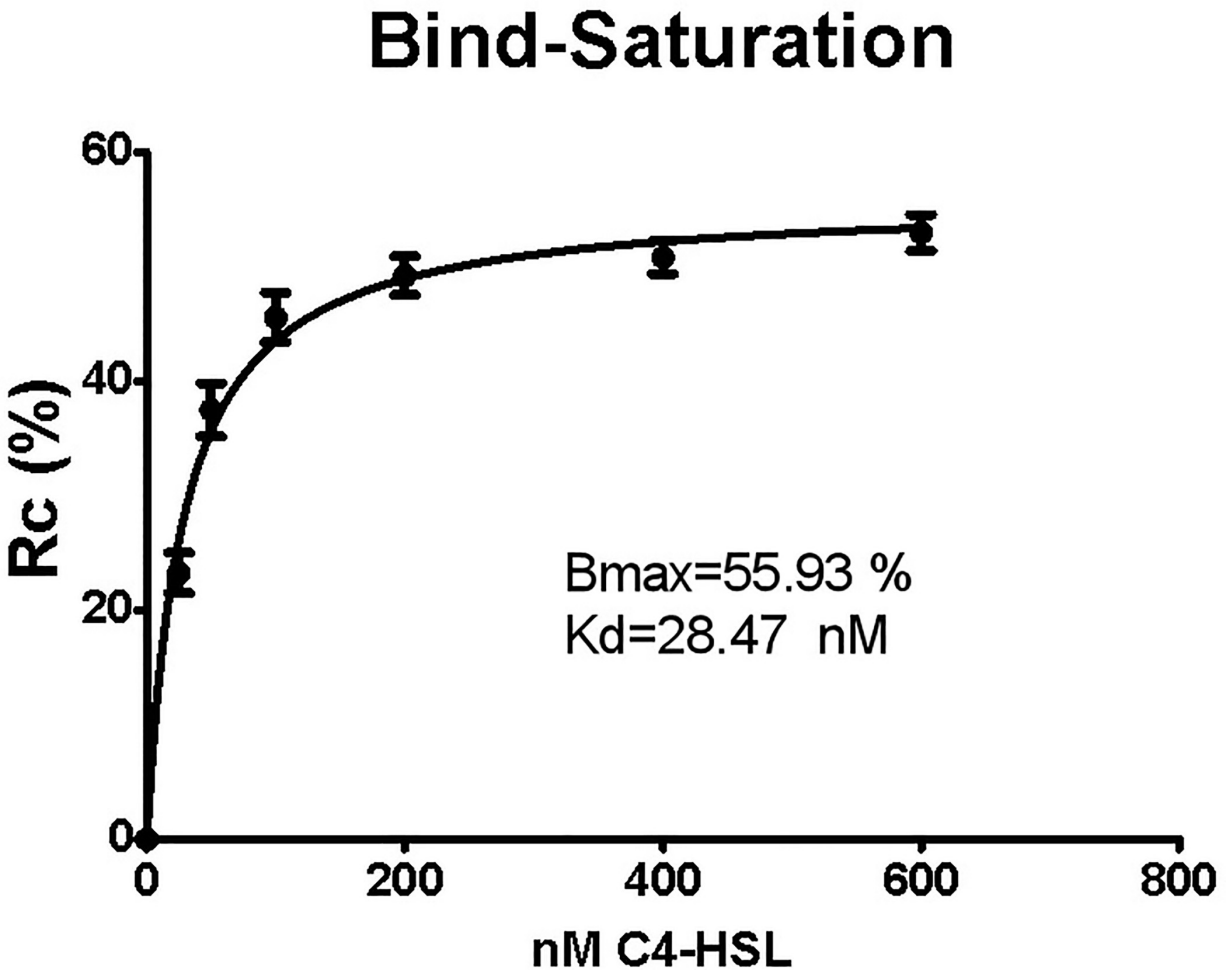
The dissociation constant of aptamer binding to C4-HSL by saturation binding model.

As shown in Fig 3 and Table 4, the Kd value reached 28.47 nM, indicating aptamer binding to C4-HSL.The structural analog of C4-HSL, 2(5H)-furanone, can be used as a competitor for C4-HSL to test the specificity of the aptamers. As shown in Table 4, even at the highest concentration of 600 nM, the structure-switching rate caused by the aptamer in combination with 2(5H)-furanone approximated 2%, which is 1/25 that of the combination with C4-HSL.

**Table 4:** Affinity analysis by saturation site binding assay and specificity analysis.

	C4-HSL					2(5H)-furanone			
	nM	test1	test2	R_*C*1_ (%)	R_*C*2_ (%)	test1	test2	R_*C*1_ (%)	R_*C*2_ (%)
0	0	174.03	173.87	0.00	0.00	-	-	-	-
1	25	264.62	279.21	21.52	25.03	-	-	-	-
2	50	322.19	341.69	35.20	39.87	-	-	-	-
3	100	356.77	374.76	43.41	47.73	-	-	-	-
4	200	374.12	388.25	47.54	50.93	-	-	-	-
5	400	381.96	393.91	49.40	52.28	176.29	176.19	0.54	0.51
6	600	390.27	403.58	51.37	54.57	182.44	183.62	2.00	2.28
*F*_*C*50_ = 512.03 *F*_*Q*_ = 91.11				*F*_*C*50_ − *F*_*Q*_ = 420.92					

### Bacterial growth inhibition test by aptamers

**Fig 4:**
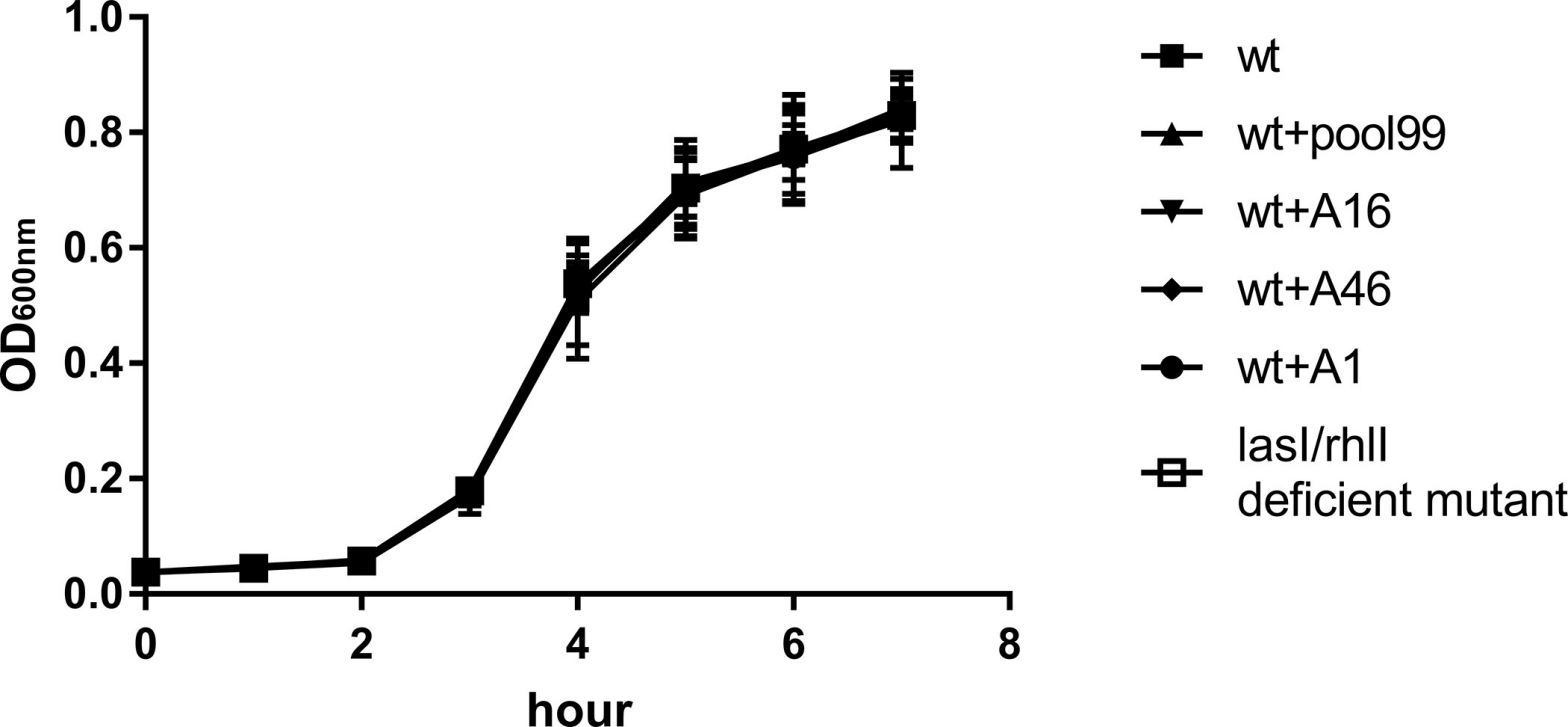
The dissociation constant of aptamer binding to C4-HSL by saturation binding mode.

The growth curves of 6 groups showed that the density of bacteria entered the exponential phase after about 2 h, and entered the growth plateau after about 5 h (Fig 4 and Table 5). No significant difference was observed among the data set of the OD_600nm_ difference between the untreated wt group and the others by one-way ANOVA, indicating that the aptamers caused no effect on bacterial growth.

**Table 5:** The OD_600nm_ of aptamers and *P*. *aeroginosa* co-incubated in liquid medium at different time points (M±s).

hour	wt	wt+pool99	wt+A16	wt+A46	wt+A1	lasI/rhlI deficient mutant
0	0.038±0.001	0.037±0.001	0.038±0.001	0.038±0.002	0.038±0.002	0.038±0.001
1	0.045±0.006	0.045±0.006	0.048±0.006	0.046±0.003	0.044±0.004	0.045±0.004
2	0.054±0.008	0.056±0.007	0.057±0.006	0.059±0.003	0.059±0.008	0.057±0.006
3	0.169±0.03	0.172±0.017	0.168±0.015	0.18±0.017	0.181±0.013	0.179±0.018
4	0.508±0.10	0.524±0.093	0.526±0.039	0.532±0.022	0.537±0.050	0.538±0.038
5	0.691±0.075	0.713±0.058	0.705±0.051	0.696±0.056	0.703±0.070	0.704±0.084
6	0.762±0.086	0.765±0.048	0.771±0.094	0.768±0.074	0.757±0.076	0.771±0.027
7	0.821±0.083	0.841±0.052	0.833±0.016	0.825±0.035	0.825±0.043	0.830±0.046

### Observing the biofilm formation inhibition by the aptamer via ESEM

**Fig 5:**
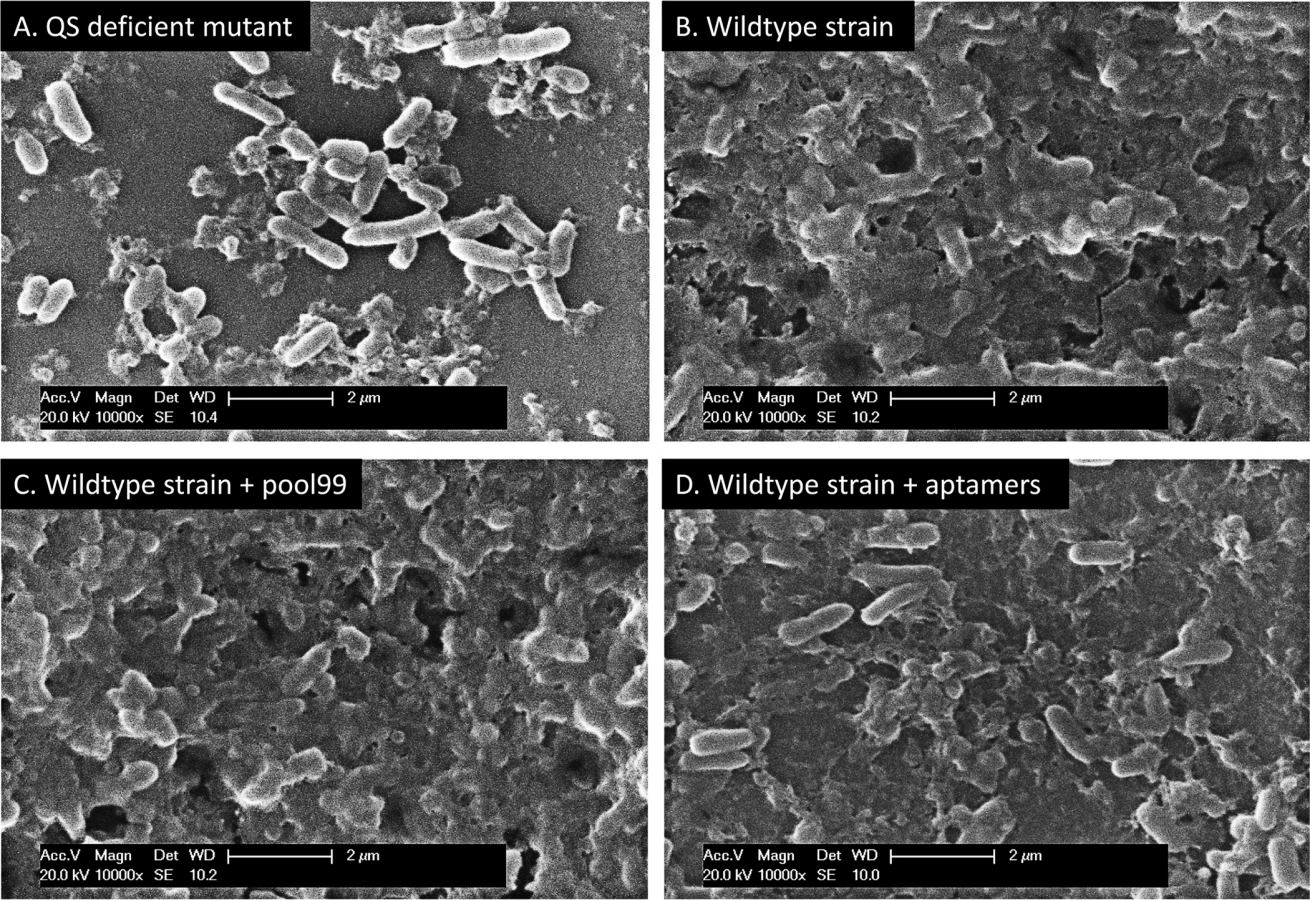
Inhibition of biofilm formation by the aptamers in vitro. A, B, C, and D represent the results of the first, second, third, and fourth groups, respectively.

In this study, polyvinyl chloride plastics were used as the solid substrate. The *P*. *aeruginosa* strains on the carriers were cultivated in the minimal medium (SB-LB medium) (20%) with aptamers for 7 days. In the whole process, the concentration of aptamers was maintained at 40 nM. Fig 5 shows the results from ESEM.

As shown in the Fig 5, as captured by ESEM, the biofilm formed by the deficient mutant was rare, and the biofilms exhibited an island-like distribution and lack of connection with each other. However, the biofilm formed by the wild type strain treated with the random Pool_99_ library was extremely thick and solid. In contrast to the biofilm of the deficient strain, this biofilm was fully fused and completely buried the cells. It was extremely similar to the biofilm formed by the wild type strain with only few differences which were more pores, channels, cracking, and collapse in the biofilm of the wild type strain. These differences implied that the biofilm formed by the wild type strain treated with the random Pool_99_ library was more stable than that formed by the wild type strain without any treatment, and that the strain had not progressed into the exfoliating and spreading stage, which may be attributed to utilization of the exogenous DNA by the strain. Compared with the wild type group and the Pool_99_ treated group, the biofilm formation in the aptamer treated group was obviously inhibited, where the biofilm was very tenuous, loose, and smooth, was remarkably inhibited. In addition, the biofilms had a mesh distribution and no fusion with each other in any position.As a result, most of the cells were not surrounded by the biofilm.

### Quantitative determination of the biofilm by the crystal violet staining method

**Fig 6:**
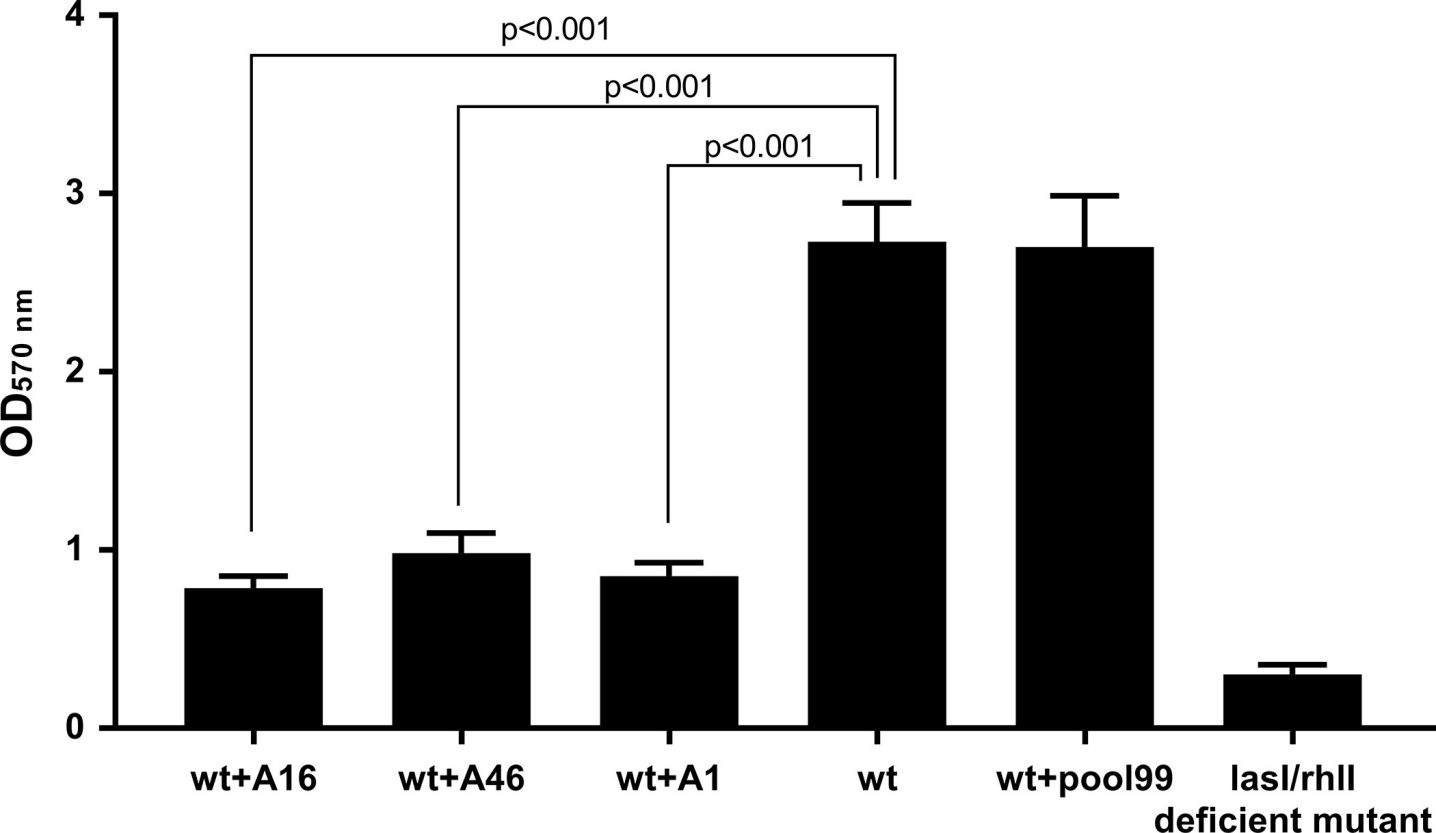
Amount of the biofilm in the different groups (OD570) by crystal violet staining.

Fig 6 shows the OD at 570 nm (OD570) of the different groups with 12 repetitions after biofilm formation *in vitro* and the dye-eluted procedure. In this study, the biofilm of the cultures in the U-type 96-well plate was quantified. Data showed (Fig 6) the lowest OD in the deficient strain group, the ODs in the wt strain group and wt strain groups treated with Pool99 were almost equal, whereas that in the wt strain group treated with every aptamer was dramatically lower than that in the no-treatment group as per one-way ANOVA. These results coincide with those observed by ESEM.

### Predicting the secondary structure of the aptamers

**Fig 7:**
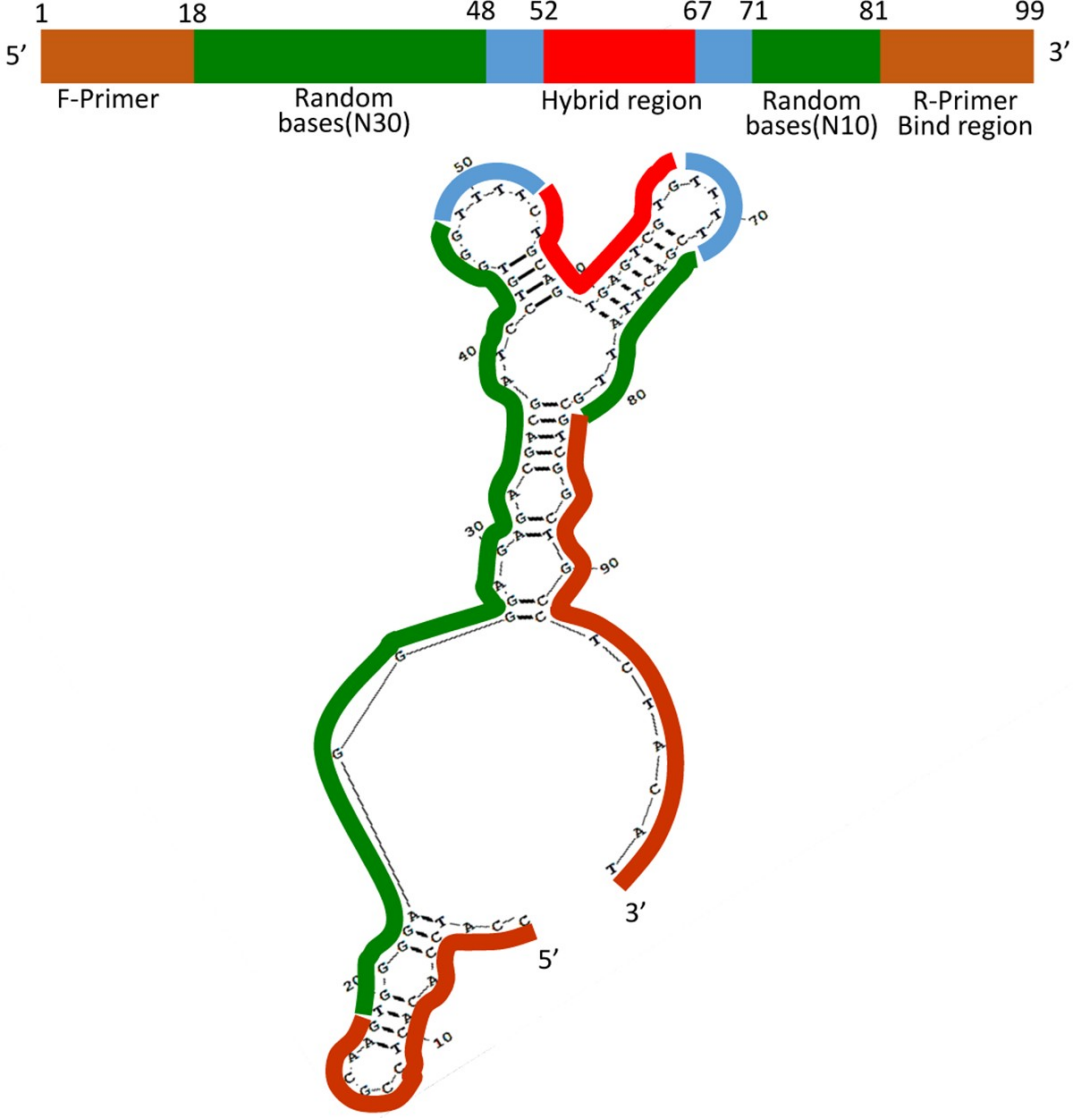
Sources of secondary structures in the aptamer.

Based on the principle that similar structures determines similar functions in biological macromolecules, the possible secondary structure of all aptamers was analyzed using the Multilign in the software RNAstructure 5.6. Then the most potential secondary structure, a Y-shaped unit similar to the Fab segment of a monoclonal antibody, was determined (Fig 7).

### Computer aided prediction of 3dRNA

**Fig 8:**
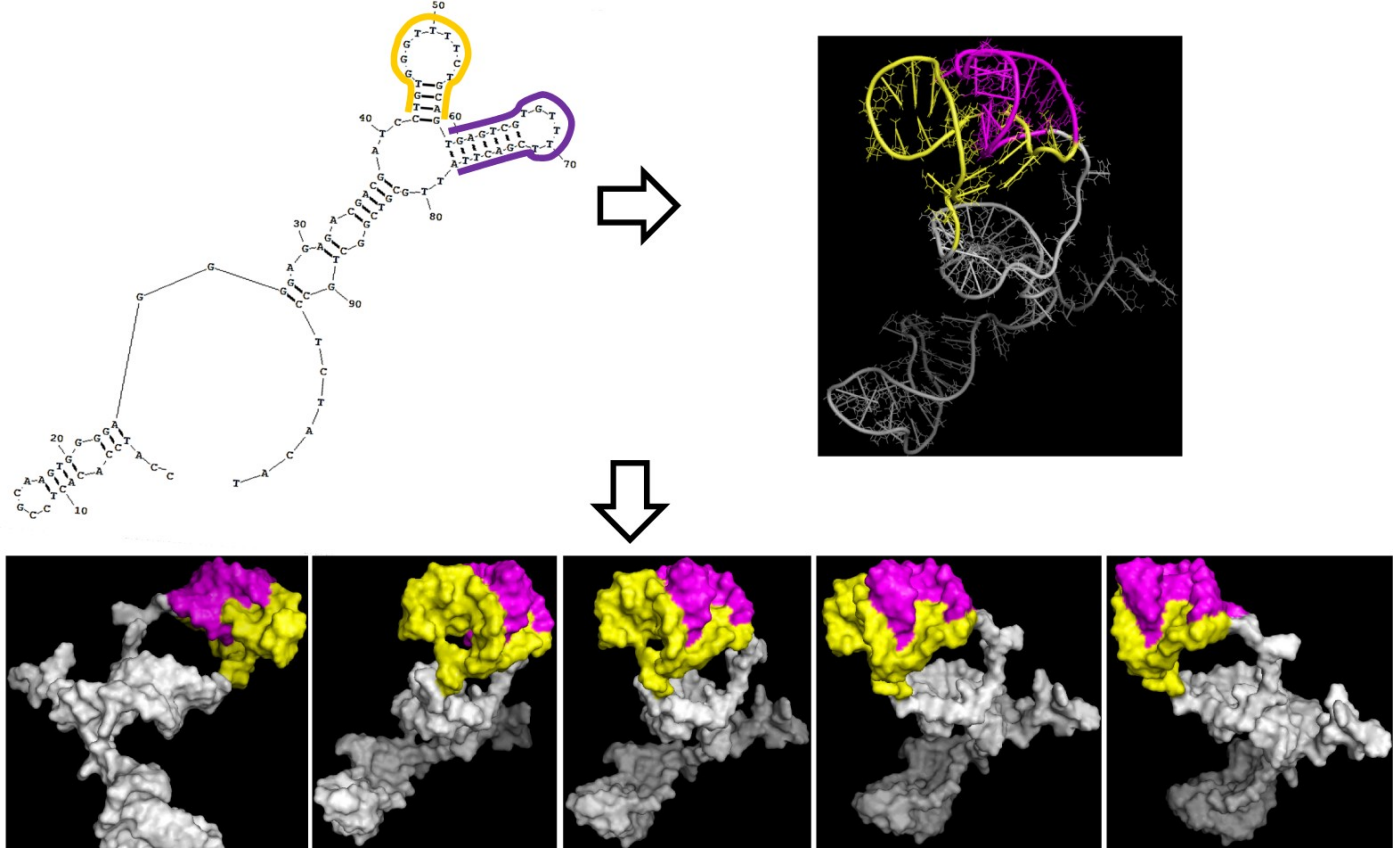
Analysis of the secondary and tertiary structure of A16 (family IV). The yellow represents the branch near the 5ʹ side in the Y-structure with the nt at the 42–58, while the purple represents the branch near the 3ʹ side in the Y-structure with the nucleotides located from 59–78.

Considering aptamer A16 in family IV as an example, the tertiary structure was simulated *de novo* with the aid of software 3dRNA. As shown in Fig 8, gray represents the skeleton of the A16. The yellow portion the branch near the 5’ side in the Y-structure with nt in 42–58 positions, whereas the purple area represents the branch near the 3ʹ side in the Y-structure with nt located at the 59–78 position. The tertiary structure simulation showed that, through screwing-crimping-winding, the Y type structure in the A16 secondary structure forms a complex tertiary structure with numerous channels and pores.

## Discussion

In clinical practice, curing *Pseudomonas aeruginosa* biofilm presents extreme difficult as most of the bacteria are encapsulated in the polysaccharide matrix. In addition to the difficulty encountered by antibiotics in entering the film, which leads to the extremely low concentration of local antibiotics to kill bacteria or inhibit their proliferation, but also a low-oxygen and nutrient-free environment also forms inside the biofilm, thus promoting the reduction of bacterial metabolism when the biofilm is formed. The bacteria can only survive by depending on the limited water and nutrients transported by the biofilm pores. In addition, most of the gene expressions of bacteria, such as numerous virulence factors, are down-regulated to avoid host immune response and reduce the sensitivity to antibiotics to protect the population. As it matures, the biofilm will locally collapse and fall off the basement membrane, causing the bacteria to migrate to and colonize other parts.

A few studies have shown that the biofilm could be inhibited through interfering with QS[9, 10, 19]. Therefore, this study intended to use aptamers to block the inducing molecules of QS, cut off signal communication among bacteria, and explore the possibility of aptamers in inhibiting biofilm formation, because when compared with antibodies, aptamers show an advantage over the target types, particularly a small molecule compound with no immunogenity. Moreover, once the candidate aptamer is obtained, various advantages, such as ease of preparation, low cost, and easy storage, will be obtained in preparation of aptamer drugs. One of the inducing molecules of QS in *Pseudomonas aeruginosa*, C4-HSL, exactly matches these characteristics. In return, the aptamers can block this molecule well, but the antibodies prove powerless against it.

The techonology of structural switching based on the principle of fluorescence resonance energy transfer has been available in the field of aptamer studies [22–30]. In this study, we improved the key point of this technology by attaching fluorophores to the microspheres as shown in Fig 1, so that the flow microsphere technology in Luminex^TM^100 can be used to quickly and accurately detect enhanced fluorescence after structural switching for monitoring the efficiency of the SELEX process. This improved technology enables simultaneous screening and efficiency monitoring of SELEX technology. Results confirmed that this technique is beneficial for improving the screening efficiency due to the less SELEX rounds (Fig 2), obtaining more identical sequences in final products (Table 3).

The aptamers screened in this study showed a higher affinity and specificity (Fig 3, Table 4), and caused no effect on the normal proliferation of bacteria (Fig 4). In vitro experiments have shown that these aptamers can inhibit the formation of biofilms of *P*. *aeruginosa* to a considerable extent (Figs 5 and 6). Based on these results, we could reasonably predict that the aptamers in this study could be used for the development of drugs and detection reagents after further optimization in the future.

Simulating the secondary and tertiary structure of aptamers not only helps to confirm the validity of the screening method, but also to further optimize the understanding of the possible sites where aptamers bind to the target. The Mutilign module of RNAstructure is based on the principle of structural conservation, which is conducive to the correct simulation of secondary structure [30–33]. As shown in Fig 7, in the middle of aptamer, the fixed sequence designed to hybridize to the capture sequence immobilized on the microsphere, is part of the Y-shaped structure. Independent computer-aided tertiary structure prediction by 3dRNA software [34, 35], showed a tertiary structure similar to the Y-shaped structure, indicating that the Y-shaped structure may be highly related to the binding site of C4-HSL. The structure must be further optimized to better implement the subject of this study.

## Supporting information

S1 Appendix The sequences of all 47 aptamers. (XLSX)
